# JNK signaling is the shared pathway linking neuroinflammation, blood–brain barrier disruption, and oligodendroglial apoptosis in the white matter injury of the immature brain

**DOI:** 10.1186/1742-2094-9-175

**Published:** 2012-07-17

**Authors:** Lan-Wan Wang, Yi-Fang Tu, Chao-Ching Huang, Chien-Jung Ho

**Affiliations:** 1 Institute of Clinical Medicine, National Cheng Kung University College of Medicine, 35 Hsiao-Tung Road, North District, 704, Tainan, Taiwan; 2 Department of Pediatrics, Chi Mei Medical Center, 901 Chung-Hua Road, Yung-Kang Disctrict, 710, Tainan, Taiwan; 3 Departments of Emergency Medicine, National Cheng Kung University College of Medicine and Hospital, 138 Sheng-Li Road, 704, Tainan, Taiwan; 4 Departments of Pediatrics, National Cheng Kung, University College of Medicine and Hospital, 138 Sheng-Li Road, 704, Tainan, Taiwan

**Keywords:** Apoptosis, Blood–brain barrier damage, Immature brain, JNK, Microglia, Neuroinflammation, Oligodendrocyte progenitor, Tumor necrosis factor-alpha, White matter injury

## Abstract

**Background:**

White matter injury is the major form of brain damage in very preterm infants. Selective white matter injury in the immature brain can be induced by lipopolysaccharide (LPS)-sensitized hypoxic-ischemia (HI) in the postpartum (P) day 2 rat pups whose brain maturation status is equivalent to that in preterm infants less than 30 weeks of gestation. Neuroinflammation, blood–brain barrier (BBB) damage and oligodendrocyte progenitor apoptosis may affect the susceptibility of LPS-sensitized HI in white matter injury. c-Jun N-terminal kinases (JNK) are important stress-responsive kinases in various forms of insults. We hypothesized that LPS-sensitized HI causes white matter injury through JNK activation-mediated neuroinflammation, BBB leakage and oligodendroglial apoptosis in the white matter of P2 rat pups.

**Methods:**

P2 pups received LPS (0.05 mg/kg) or normal saline injection followed by 90-min HI. Immunohistochemistry and immunoblotting were used to determine microglia activation, TNF-α, BBB damage, cleaved caspase-3, JNK and phospho-JNK (p-JNK), myelin basic protein (MBP), and glial fibrillary acidic protein (GFAP) expression. Immunofluorescence was performed to determine the cellular distribution of p-JNK. Pharmacological and genetic approaches were used to inhibit JNK activity.

**Results:**

P2 pups had selective white matter injury associated with upregulation of activated microglia, TNF-α, IgG extravasation and oligodendroglial progenitor apoptosis after LPS-sensitized HI. Immunohistochemical analyses showed early and sustained JNK activation in the white matter at 6 and 24 h post-insult. Immunofluorescence demonstrated upregulation of p-JNK in activated microglia, vascular endothelial cells and oligodendrocyte progenitors, and also showed perivascular aggregation of p-JNK-positive cells around the vessels 24 h post-insult. JNK inhibition by AS601245 or by antisense oligodeoxynucleotides (ODN) significantly reduced microglial activation, TNF-α immunoreactivity, IgG extravasation, and cleaved caspase-3 in the endothelial cells and oligodendrocyte progenitors, and also attenuated perivascular aggregation of p-JNK-positive cells 24 h post-insult. The AS601245 or JNK antisense ODN group had significantly increased MBP and decreased GFAP expression in the white matter on P11 than the vehicle or scrambled ODN group.

**Conclusions:**

LPS-sensitized HI causes white matter injury through JNK activation-mediated upregulation of neuroinflammation, BBB leakage and oligodendrocyte progenitor apoptosis in the immature brain.

## Background

Spastic cerebral palsy develops in 5 to 10% of the survivors among very preterm infants (less than 32 weeks of gestation age) [1,2]. Cerebral white matter injury is the major form of brain injury and the leading cause of cerebral palsy in children who are born very prematurely [1,2]. The neuropathologic hallmark of white matter injury in preterm infants includes a multitude of activated microglia and macrophages that produce pro-inflammatory cytokines at early stage, and focal and diffuse white matter lesions along with astrocytosis and hypomyelination at late stage [1,2].

Epidemiological observations show that hypoxic-ischemia (HI) and infection are the two major risk factors of white matter injury and cerebral palsy in very preterm infants [3-5]. Clinical studies have implicated the potentiating effect of infection on HI in preterm infants [6-8]. Animal studies have also shown that pre-exposure to systemic lipopolysaccharide (LPS) sensitized HI injury in the cerebral cortex and white matter of postpartum (P) day 7 or 8 rodent pups, where brain maturation status is equivalent to 32 to 34 weeks of gestation of preterm infants [9-11]. The O4-positive oligodendrocyte progenitors are the target cells of damage during the window of vulnerability for white matter injury in premature infants at 23 to 32 weeks of gestation [12]. Comparing the timing of human and rodent oligodendroglial lineage progression, the predominance of pre-myelinating oligodendrocytes in P2 rat pups (brain maturation status equivalent to very preterm infants less than 30 weeks) coincides with the high-risk period of white matter injury in very preterm infants [13]. Our previous study in P2 rat pups demonstrated that LPS or 90-minute HI (a sub-threshold duration for P2 pups) alone caused no significant injury in the cortex or white matter, whereas selective white matter injury could only be induced by the combination of the two [14]. The findings suggest that LPS sensitizes HI, and selectively causes white matter injury in the immature brain.

The major target of ischemic-reperfusion injury in the cerebral cortex is the neurovascular unit, which is composed of neurons, microglia and microvessels [15]. Neuronal apoptosis, microglia activation and microvascular damage, in other words blood–brain barrier (BBB) disruption, have been linked with the severity of HI cortical neuronal injury in P7 to P10 rat pups [16-19]. Similar to the framework of the “neurovascular unit” in the cerebral cortex [15], microglia, oligodendrocyte progenitors and microvascular endothelial cells may form a closely inter-related “oligodendrovascular unit” in the white matter, which may be the major target of white matter injury in the preterm infants. During detrimental insults in the immature brain, activated microglia may exacerbate white matter injury through production of pro-inflammatory cytokines, such as TNF-α [1,20]. The damaged microvessels may recruit activated leukocytes into the injured white matter through the disrupted BBB, resulting in sustained activation of microglia, which in turn further damage the white matter through prolonged production of inflammatory cytokines [21]. Since microglia, vascular endothelial cells and oligodendrocytes may closely interact with each other in the white matter, there may be a common signaling mechanism linking neuroinflammation, BBB disruption and oligodendroglial progenitor cell apoptosis in the white matter injury of the immature brain.

c-Jun N-terminal kinases (JNK) are important stress-responsive kinases that are activated by various forms of insults, including ischemia [22]. JNK activation precedes cell death by inflammation and apoptosis in many cell types [23]. Activation of JNK signaling leads not only to pro-inflammatory cytokine production, but also to cell death via intrinsic/extrinsic apoptotic pathways [22,24-28]. *In vitro* studies show that JNK signaling is the predominant pathway for cytokine production from LPS-stimulated or hypoxia-exposed microglia [29,30]. JNK signaling also plays a crucial role in subarachnoid hemorrhage-associated BBB disruption, and stress-induced apoptosis of cerebral endothelial cells and oligodendrocyte progenitors [31-33]. *In vivo* studies demonstrated early and lasting JNK activation after cerebral ischemia [34,35]. Our previous study in P7 rat pups showed that neonatal overweight increased HI-induced neuronal apoptosis, microglial activation and BBB damage in the cerebral cortex, and aggravated cortical damage through JNK hyperactivation [18]. However, it remains unclear whether JNK activation is the common pathogenic mechanism in the “oligodendrovascular unit” leading to white matter damage in the immature brain of P2 rat pups.

Using an established model of LPS-sensitized HI white matter injury in P2 rat pups [14], we hypothesized that JNK signaling is the shared pathway linking neuroinflammation, microvascular endothelial cell damage and BBB breakdown, and apoptosis of oligodendroglial precursor cells in the white matter injury of the immature brain.

## Methods

### A selective white matter injury model in P2 rat pups induced by lipopolysaccharide-sensitized hypoxic-ischemia

The animal study was approved by the Animal Care Committee at National Cheng Kung University. Sprague–Dawley rat pups were housed under standard condition with a 12/12-h light/dark cycle. We first injected P2 rat pups intraperitoneally with 0.05 mg/kg LPS (*Escherichia coli* 0111:B4; Sigma-Aldrich, St Louis, MO, USA) or pyrogen-free normal saline (NS). Neuropathological examinations performed on P11 showed that, compared with the NS-treated group, the LPS-treated pups had no significant injury in the cortex ( Additional file 1: Figure 1A) and white matter ( Additional file 1: Figure 1B). The LPS-treated pups also showed no evidence of microglial activation and BBB breakdown in the white matter ( Additional file 1: Figure 1C). These findings suggested low-dose LPS did not cause damage in the cortex or upregulate neuroinflammation and BBB disruption in the white matter of P2 rat pups.

We then injected P2 pups with LPS (0.05 mg/kg) or NS 3 h before HI, as described previously [14]. Pups were randomly assigned to three different groups: control (NS without HI), NS + HI (NS injected 3 h before HI), and LPS + HI (LPS injected 3 h before HI). To avoid LPS-induced body temperature changes, the rat pups were returned to their dams after injection, and housed in an incubator to maintain body temperature at 33 to 34 °C before HI. HI was then induced by ligation of the right carotid artery followed by hypoxia [36]. The right common carotid artery was permanently ligated under 2.5% halothane anesthesia. After surgery, the pups were returned to an incubator for a 1-h recovery. They were then placed in airtight 500 mL containers partially submerged in a 36 °C water bath, and humidified 6.5% oxygen was kept at a flow rate of 3 L/minute for 90 minutes. Following hypoxia, pups were returned to their dam.

### Pharmacological inhibition of JNK

AS601245, a highly specific JNK inhibitor, blocks JNK activity by binding to its ATP-binding site [37]. The P2 pups were randomly assigned to three different groups: (1) control group without being exposed to LPS + HI; (2) intraperitoneal injection of vehicle (DMSO, Sigma-Aldrich) 30 minutes before and immediately after LPS + HI; and (3) intraperitoneal injection of AS601245 20 or 40 mg/kg (Alexis Biochemicals, Lausen, Switzerland) 30 minutes before and immediately after LPS + HI. The dose of AS601245 used in this study was modified from the study by Carboni and colleagues [37].

### Knockdown of JNK gene expression by antisense oligodeoxynucleotides

P2 pups were intracerebroventricularly infused with JNK antisense or scrambled oligodeoxynucleotides (ODN) into the right cerebral hemisphere using a 30-gauge needle on a 10 μL Hamilton syringe with an infusion rate of 1 μL/minute, as previously described [38]. The injection location was 2.0 mm posterior to and 1.5 mm lateral to the bregma and 2.0 mm beneath the skull surface. The first ODN (100 pmol in 1 μL) were injected 30 minutes before LPS + HI, and the second ODN (100 pmol in 1 μL) given immediately after LPS + HI. The sequences of the JNK antisense were 5’-TTT CTT CAT GAA YTC-3’, and the scrambled ODNs were 5’-GTC TTG AAC TTC CCG -3’. Based on the mRNA sequences for rat JNK isoforms (Genebank accession number JNK1, XM_341399; JNK2, NM_017322; JNK3, NM_012806), the antisense sequence matched the rat JNK1-3 cDNA sequences, while the scrambled ODN showed no significant matches. The pups that were not exposed to LPS + HI served as the control group. The white matter tissues were collected for Western blot analyses at 3, 6 and 12 h after the second ODN injection.

### Western blot analysis

The temporal profile of JNK activation after LPS + HI was assessed using Western blot analysis. Ipsilateral cerebral white matter tissues were homogenized in cold lysis buffer, and the protein concentrations determined using a Bio-Rad Protein Assay kit (Bio-Rad Laboratories, Hercules, CA, USA). Samples (50 μg) were separated using 10% SDS-PAGE and blotted onto polyvinylidene fluoride membranes. Membranes were incubated with primary antibodies, and immunoreactivity was detected by horseradish-conjugated secondary antibody and visualized using enhanced chemiluminescence. The following primary antibodies were used: anti-JNK (1:1000; Cell Signaling, Danvers, MA, USA), anti-phospho-JNK (Thr183/Tyr185) (1:1000; Cell Signaling), and anti-actin (1:5000; Millipore, Billerica, MA, USA). Western blot signals were quantified by scanning with a ScanJet scanner (Hewlett Packard, Palo Alto, CA, USA), and the band intensity was analyzed using an imaging software (ImagePro Plus 6.0; Media Cybernetics, Bethesda, MD, USA).

### *In vitro* kinase assay for JNK activity

We compared JNK activity between the vehicle-treated and AS601245-treated pups at 6 and 24 h post-insult. JNK activity was measured using a specific kit (Cell Signaling), and glutathione S-transferase-Jun (1–79) fusion peptides served as the substrate for JNK as previously described [18]. In brief, white matter tissue lysates (200 μg) were incubated overnight at 4 °C with glutathione S-transferase-Jun fusion protein beads. After washing, the beads were resuspended in kinase buffer containing ATP, and the kinase reaction was allowed to continue for 30 minutes at 30 °C. Reactions were stopped by adding polyacrylamide gel electrophoresis sample loading buffer. Proteins were separated by electrophoresis on 10% SDS-PAGE, transferred onto polyvinylidene fluoride membrane, and incubated with phospho-c-Jun (Ser63) antibody (1:1000; Cell Signaling). Immunoreactivity was detected using enhanced chemiluminescence (Amersham, Piscataway, NJ, USA).

### Immunohistochemistry

The pups were sacrificed and perfused for cryosections at 6 and 24 h post-insult on P2. The brains were post-fixed in ice-cold 4% paraformaldehyde overnight, dehydrated using 30% (w/v) sucrose in PBS for 2 days, and coronally sectioned (20-μm thick) from the genu of the corpus callosum to the end of the dorsal hippocampus. Four coronal sections, two at the level of the striatum (0.26 mm and 0.92 mm posterior to the bregma) and another two at the levels of the dorsal hippocampus (3.14 mm and 4.16 mm posterior to the bregma) selected according to a rat brain atlas [39], were assessed for each brain.

Immunohistochemistry for phospho-JNK (p-JNK) was performed at 6 h and 24 h post-insult, while staining for microglial activation, TNF-α, IgG, and cleaved caspase 3 was performed at 24 h post-insult. IgG extravasation was used as an indicator of BBB permeability [40]. The specific primary antibodies used included rabbit polyclonal anti-p-JNK (1:100; Cell Signaling), mouse anti-rat ED1 (microglia marker; 1:100; Millipore), rabbit polyclonal anti-rat TNF-α (1:100; Bender MedSystems, Vienna, Austria), horseradish peroxidase-conjugated goat anti-rat IgG (1:200; Millipore) and rabbit polyclonal anti-cleaved caspase 3 (1:100; Cell Signaling). Biotinylated secondary antibodies included anti-mouse IgG and anti-rabbit IgG (all 1:200; Pierce Biotechnology, Rockford, IL, USA). Biotin-peroxidase signals were detected using 0.5 mg/mL 3’3’-diaminobenzidine (DAB)/0.003% H_2_O_2_ (Dako, Carpinteria, CA, USA) as a substrate. Results were recorded using a microscope (BX51; Olympus, Tokyo, Japan).

### Assessment for white matter injury

The brains were prepared in paraffin sections for pathological examinations on P11. The brains were removed and post-fixed in 4% paraformaldehyde at room temperature for 48 h, dehydrated through graded alcohols and embedded in paraffin, and then coronally sectioned (10-μm thick) from the genu of the corpus callosum to the end of the dorsal hippocampus. Myelin basic protein (MBP) staining for myelination and glial fibrillary acidic protein (GFAP) staining for astrogliosis in the white matter were used as markers of white matter injury. Four coronal sections, two at the level of the striatum (0.26 mm and 0.92 mm posterior to the bregma) and another two at the level of the dorsal hippocampus (3.14 mm and 4.16 mm posterior to the bregma) according to a rat brain atlas [39], were assessed for each brain. Paraffin-embedded sections were deparaffinized and hydrated through graded alcohols. Endogenous peroxidases were eradicated for 30 minutes in 0.3% H_2_O_2_ in methanol. Heat-induced antigen retrieval was subsequently performed using 10 nmol/L citrate buffer (pH = 6.0) for 10 minutes in a microwave oven. After permealization and blocking of non-specific binding, sections were first incubated at 4 °C overnight with rat anti- MBP monoclonal antibody (1: 100; Millipore) or rabbit polyclonal anti-GFAP antibody (1: 800; Millipore), rinsed, and then incubated for 1 h at room temperature with goat anti-rat (1:200; Santa Cruz Biotechnology, Santa Cruz, CA, USA) or anti-rabbit (1:300; Pierce Biotechnology) biotinylated secondary antibodies. Positively-stained cells were visualized using avidin-biotin-peroxidase complex amplification (Pierce Biotechnology) with diaminobenzidine tetrahydrochloride detection.

MBP expression was graded in three regions within the white matter in each hemisphere of each section using a 4-point scoring system - 0, loss of processes and complete loss of capsule; 1, loss of processes with thinning or breaks in capsule; 2, complete loss of processes with intact capsule; 3, partial loss of processes; 4, no MBP loss - as previously described [14]. The scores of each region were summed to obtain a total score (range 0 to 12) for each hemisphere. Each section had a total MBP score in the ipsilateral and contralateral hemisphere, respectively. Observers, blind to the treatment conditions, assessed the degrees of white matter injury.

### Quantitative analysis of immunohistochemical staining

Measurement of MBP scores, the number of ED1 and cleaved caspase 3-positive cells, and the integrated optical density (IOD) of p-JNK, TNF-α, IgG and GFAP signals were respectively analyzed as previously described [41], using an imaging software (ImagePro Plus 6.0; Media Cybernetics). Measurement was performed at 400× magnification per visual field (0.0356 mm^2^) for cleaved caspase 3-positive cell numbers, 100× magnification per visual field (0.579 mm^2^) for MBP scores, and 200× magnification per visual field (0.145 mm^2^) for p-JNK, TNF-α, IgG and GFAP signals, and ED1-positive cell numbers. Three visual fields in the medial, middle and lateral areas of the white matter in each hemisphere per section and four sections per brain were analyzed and averaged, respectively. The mean IOD values in the white matter of the ipsilateral and contralateral hemispheres of each experimental group were compared to those of the control group to obtain the relative IOD ratios.

### Immunofluorescent staining

Immunofluorescence was performed at 6 and 24 h post-insult. After blocking (1× PBS, 2% normal goat serum and 0.1% Triton X-100) for 1 h, the sections were incubated overnight at 4 °C with a mixture of two of the following primary antibodies: mouse anti-rat ED1 (1:100; Millipore), mouse monoclonal anti-O4 IgM (1:100; Millipore), mouse monoclonal anti-rat endothelial cell antigen-1 (RECA-1; 1:100; Abcam, Cambridge, MA, USA), rabbit polyclonal anti-p-JNK (1:100; Cell Signaling), mouse monoclonal anti-p-JNK (1:100; Cell Signaling), rabbit polyclonal anti-p-c-Jun (1:100; Cell Signaling), rabbit polyclonal anti-rat TNF-α (1:100; Bender MedSystems) and rabbit polyclonal anti-cleaved caspase 3 (1:100; Cell Signaling). The sections were washed three times with 0.1 M PBS and then incubated with Alexa Fluor 594 anti-mouse IgG/IgM or Alexa Fluor 488 anti-rabbit IgG (all 1:400; Invitrogen, Grand Island, NY, USA) for 1 h at room temperature. Nuclei were visualized with 4',6-diamidino-2-phenylindole (DAPI). Slides were photographed for red (Alexa Fluor 594) and green (Alexa Fluor 488) fluorescence with a fluorescent microscope (E400; Nikon, Tokyo, Japan).

### Statistical analysis

Statistical significance (*P* < 0.05) was determined using Kruskal-Wallis test, and Dunn’s method was used for *post hoc* comparisons. Continuous data were presented as means ± standard error of the mean (SEM).

## Results

### Neuroinflammation, blood–brain barrier damage and cell apoptosis in association with cerebral white matter injury in rat pups after lipopolysaccharide-sensitized hypoxic-ischemia

**Figure 1:**
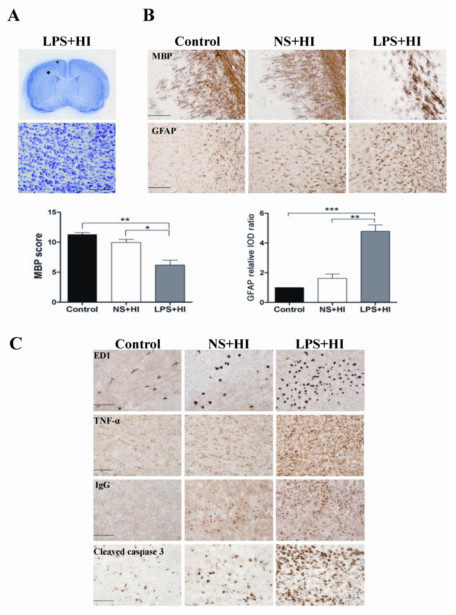
**Upregulation of neuroinflammation, blood–brain barrier damage and cell apoptosis in association with white matter injury in P2 rat pups after lipopolysaccharide-sensitized hypoxic-ischemia.** On P11 in the LPS + HI group, Nissl staining (**A**) showed no significant injury in the cortex (gross picture in the upper panel; microscopic picture in the lower panel photographed from the cortex marked with an asterisk). (**B**) Immunohistochemical staining demonstrated that the LPS + HI group had markedly decreased MBP expression and increased GFAP-positive astrogliosis in the white matter of the ipsilateral hemisphere compared to the control and NS + HI groups. (**C**) Immunohistochemistry 24 h post-insult showed that the LPS + HI but not the NS + HI group had significant increases in ED1-positive microglia, TNF-α immunoreactivities, IgG extravasation, and cleaved caspase 3-positive apoptotic cells in the white matter. Microscopic pictures of (B,C) were taken from the white matter area marked with a circle in (A). ED1, microglia marker; GFAP, glial fibrillary acidic protein; HI, hypoxic-ischemia; LPS, lipopolysaccharide; MBP, myelin basic protein; NS, normal saline; P, postpartum. Scale bar = 200 μm for MBP, 50 μm for cleaved caspase 3, and 100 μm for the others.

On P11 (9 days post-insult), Nissl staining showed no significant injury in the cerebral cortex after LPS-sensitized HI on P2 (Figure 1A). In contrast, significant white matter injury was found as evidenced by marked decreases of MBP expression and increases of GFAP (astrogliosis) in the ipsilateral hemisphere of the LPS + HI group but not of the NS + HI group (Figure 1B). Twenty-four hours after injury on P2, the LPS + HI (but not the NS + HI group) had significant increases of ED1-positive activated microglia, TNF-α expression, IgG extravasation (BBB damage) and cleaved caspase 3-positive cells in the white matter compared to the control group (Figure 1C). These findings suggested upregulation of neuroinflammation, BBB disruption and cell apoptosis in the P2 rat pup model of selective white matter injury induced by LPS + HI.

### Early and sustained JNK activation in the microglia, endothelial cells and oligodendrocyte progenitors of the white matter after lipopolysaccharide-sensitized hypoxic-ischemia

**Figure 2:**
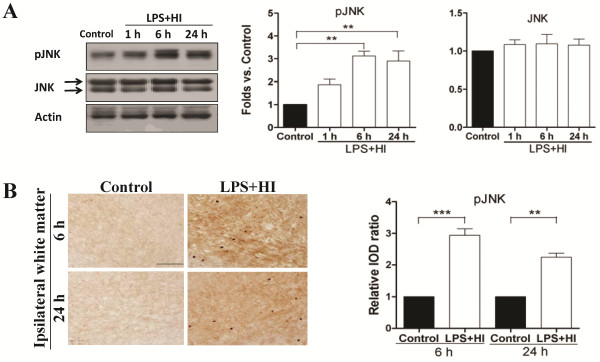
**Upregulation of JNK activation in lipopolysaccharide-sensitized hypoxic-ischemic white matter injury.** (**A**) Immunoblotting of white matter in the lipopolysaccharide (LPS) + hypoxic-ischemic (HI) group showed there was an early rise of phospho-c-Jun N-terminal kinase (p-JNK) expression at 1 h, which peaked at 6 h and persisted at 24 h post-insult. The JNK expression did not differ between the control and LPS + HI groups at various time points post-insult. (**B**) p-JNK immunohistochemistry at 6 and 24 h post-insult showed the LPS + HI group (n = 10) had significantly higher p-JNK immunoreactivities in the white matter of the ipsilateral hemisphere than the control (n = 7) groups. Scale bar =100 μm in (B). Values are means ± SEM. ***P < 0.001, **P < 0.01.

**Figure 3:**
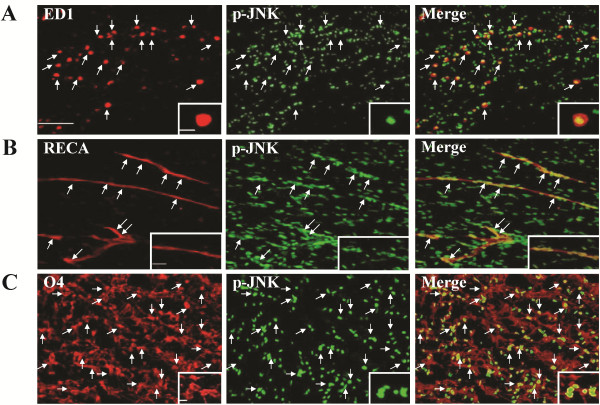
**JNK activation in microglia, vascular endothelial cells and oligodendrocyte progenitors at 6 h post-insult.** Immunofluorescence of the ipsilateral white matter in the lipopolysaccharide (LPS) + hypoxic-ischemic (HI) group showed increased phospho-c-Jun N-terminal kinase (p-JNK) expression in (**A**) ED1-positive microglia, (**B**) RECA-positive endothelial cells and (**C**) O4-positive oligodendrocyte progenitors. Scale bar = 25 μm. Inset scale bar = 5 μm in (A), 10 μm in (B) and 2.5 μm in (C).

**Figure 4:**
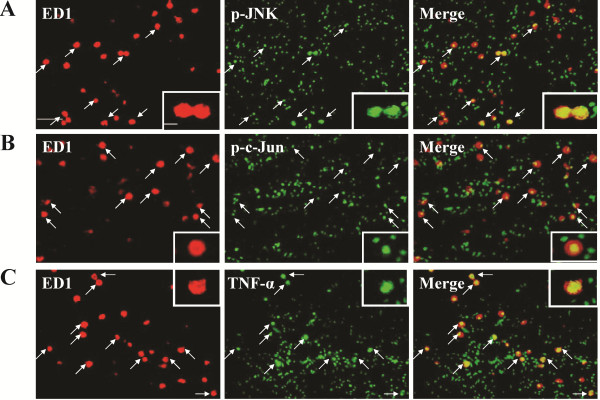
**Activated microglia expressed p-JNK, p-c-Jun and TNF-α.** Immunofluorescence of the ipsilateral white matter in the lipopolysaccharide (LPS) + hypoxic-ischemic (HI) group 24 h post-insult showed that ED1-positive activated microglia expressed phospho-c-Jun N-terminal kinases (p-JNK) (**A**) and TNF-α (**C**), and had nuclear translocation of p-c-Jun (**B**). Scale bar = 25 μm. Inset scale bar = 5 μm.

**Figure 5:**
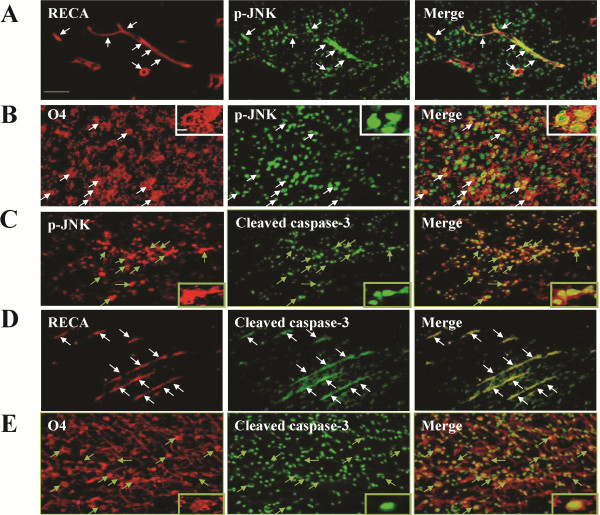
**JNK activation-mediated apoptosis in cerebral vascular endothelial cells and oligodendrocyte progenitors in the white matter after lipopolysaccharide-sensitized hypoxic-ischemia.** Immunofluorescence of the lipopolysaccharide (LPS) + hypoxic-ischemic (HI) group 24 h post-insult showed numerous phospho-c-Jun N-terminal kinase (p-JNK)-positive cells attached to or located around the microvessels in the white matter (**A**). RECA-positive endothelial cells (**A**) and O4-positive oligodendrocyte progenitors (**B**) co-expressed p-JNK. Many p-JNK-positive cells (**C**), RECA-positive endothelial cells (**D**) and O4-positive oligodendrocyte progenitors (**E**) expressed cleaved caspase 3. Scale bar = 25 μm. Inset scale bar = 2.5 μm.

Immunoblotting analyses of ipsilateral white matter demonstrated increased JNK phosphorylation at 24 h after LPS ( Additional file 1: Figure 1D), whereas JNK activation occurred early at 1 h, peaked at 6 h and persisted at 24 h post-insult in the LPS + HI group (Figure 2A). Immunohistochemical analyses confirmed that the LPS + HI group had increases of p-JNK immunoreactivities in the white matter at 6 and 24 h post-insult compared to the control group (Figure 2B). Further immunofluorescence studies showed upregulated p-JNK expression in the ED1-positive activated microglia, RECA-positive vascular endothelial cells and O4-positive oligodendrocyte progenitors in the white matter at 6 h (Figure 3A,B,C) and 24 h (Figures 4A, 5A,B) post-insult. The activated ED1-positive microglia showed nuclear translocation of p-c-Jun, the downstream signal molecule of p-JNK (Figure 4B), and also highly expressed TNF-α 24 h post-insult (Figure 4C). Characteristically, there were numerous p-JNK-positive cells attached to or located around the microvessels in the white matter (Figure 5A). Furthermore, many of the p-JNK-positive cells co-expressed cleaved caspase 3 (Figure 5C). Both vascular endothelial cells and oligodendroglial progenitor cells also co-expressed cleaved caspase 3 (Figure 5D,E), indicating these cells underwent apoptosis. These findings suggested the involvement of JNK activation in neuroinflammation, and apoptosis of endothelial cells and oligodendroglial progenitors in the white matter after LPS + HI injury.

### Pharmacological inhibition of JNK reduced neuroinflammation, blood–brain barrier damage and cell apoptosis, and protected against white matter injury after lipopolysaccharide-sensitized hypoxic-ischemia

**Figure 6:**
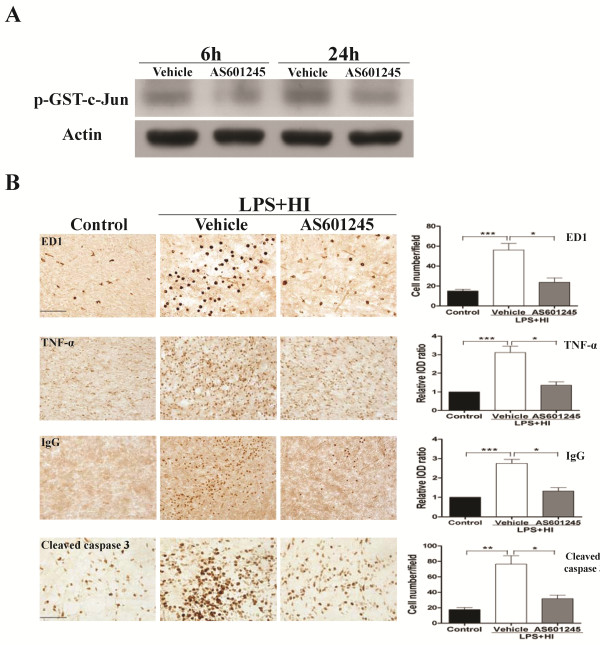
**AS601245 significantly reduced neuroinflammation, blood–brain-barrier damage and cell apoptosis after lipopolysaccharide-sensitized hypoxic-ischemic white matter injury.** (**A**) *In vitro* kinase assay of c-Jun N-terminal kinase (JNK) in the lipopolysaccharide (LPS) + hypoxic-ischemic (HI) group showed that AS601245 (40 mg/kg) effectively blocked JNK activity at 6 and 24 h post-insult compared with vehicle. (**B**) AS601245 (40 mg/kg) treatment (n = 8) significantly reduced upregulation of ED1-positive activated microglia, TNF-α immunoreactivities, IgG extravasation and cleaved caspase 3-positive cells in the white matter 24 h post-insult compared to vehicle (n = 7). Scale bar =100 μm for ED1, TNF-α and IgG; 50 μm for cleaved caspase 3. Values are means ± SEM. ***P <0.001, **P < 0.01, *P < 0.05.

**Figure 7:**
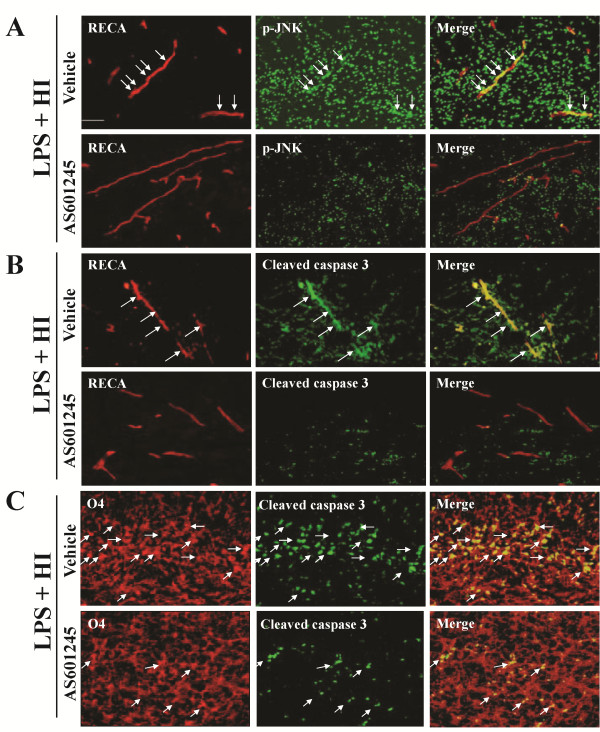
**AS601245 significantly reduced perivascular attachment of p-JNK-positive cells and cell apoptosis in the white matter 24 h post-insult.** Immunofluorescent staining in the lipopolysaccharide (LPS) + hypoxic-ischemic (HI) group showed that, compared with vehicle, AS601245 (40 mg/kg) significantly attenuated perivascular phospho-c-Jun N-terminal kinase (p-JNK)-positive cell attachment (**A**), and also decreased cleaved caspase 3-positive endothelial (**B**) and oligodendroglial cells (**C**) in the white matter. Scale bar =25 μm.

**Figure 8:**
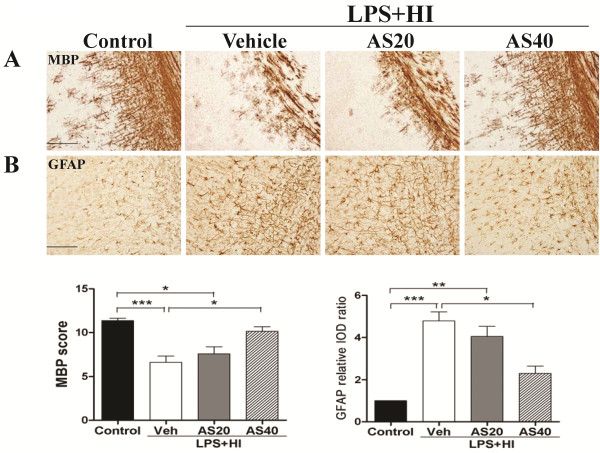
**Pharmacological inhibition of c-Jun N-terminal kinase (JNK) activity using AS601245 significantly attenuated white matter injury.** AS601245 (40 mg/kg) (n = 8) but not AS601245 (20 mg/kg) (n = 8) treatment had significantly higher myelin basic protein (MBP) (**A**) and lower glial fibrillary acidic protein (GFAP) (**B**) expression in the white matter than vehicle (n = 10) on P11 after lipopolysaccharide (LPS)-sensitized hypoxic-ischemia (HI) on P2. Scale bar = 200 μm in (A), 100 μm in (B). Values are means ± SEM. ***P < 0.001, **P < 0.01, *P < 0.05.

We then examined the protective effect of JNK inhibition on white matter injury using AS601245, an ATP-competitive inhibitor of JNK. *In vitro* kinase assay in the LPS + HI group confirmed that AS601245 (40 mg/kg) treatment significantly reduced JNK activity compared to vehicle treatment at 6 and 24 h post-insult (Figure 6A). In the LPS + HI group, AS601245 treatment significantly decreased the numbers of ED1-positive activated microglia, TNF-α immunoreactivities, BBB damage and cleaved caspase 3-positive cells in the white matter 24 h post-insult compared to vehicle treatment (Figure 6B). Further immunofluorescent staining showed that AS601245 markedly decreased the p-JNK (+) cells attached to or located around the microvessels (Figure 7A), and also greatly attenuated cleaved caspase 3 expression in vascular endothelial cells (Figure 7B) and oligodendroglial progenitor cells (Figure 7C). Compared to vehicle, AS601245 treatment on P2 at a dosage of 40 mg/kg but not 20 mg/kg in the LPS + HI group significantly preserved MBP expression (Figure 8A) and markedly attenuated astrogliosis by downregulating GFAP immunoreactivities (Figure 8B) in the white matter on P11.

### Genetic knockdown of JNK expression reduced neuroinflammation, blood–brain barrier disruption and cell apoptosis, and attenuated white matter injury after lipopolysaccharide-sensitized hypoxic-ischemia

**Figure 9:**
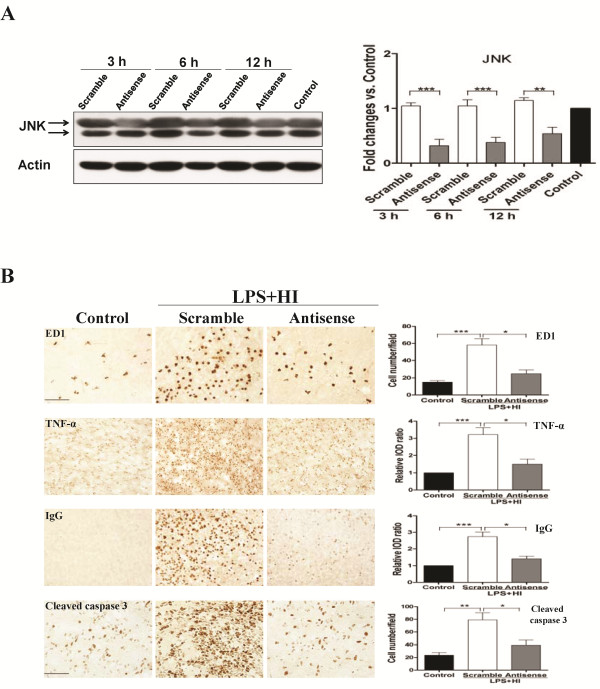
**JNK antisense oligodeoxynucleotide significantly reduced neuroinflammation, blood–brain-barrier damage and apoptosis in the white matter after lipopolysaccharide-sensitized hypoxic-ischemia.** (**A**) Immunoblotting of the white matter showed that intracerebroventricular infusion of c-Jun N-terminal kinase (JNK) antisense oligodeoxynucleotides (ODN) (n = 3) effectively suppressed JNK expression compared with scrambled ODN (n = 3) at 3, 6 and 12 h post-insult. (**B**) Antisense ODN treatment (n = 8) significantly attenuated upregulation of ED1-positive activated microglia, TNF-α immunoreactivities, IgG extravasation and cleaved caspase 3-positive cells in the white matter 24 h post-insult compared with scrambled oligodeoxynucleotide (n = 8). Scale bar =100 μm for ED1, TNF-α and IgG; 50 μm for cleaved caspase 3. Values are means ± SEM. ***P < 0.001, **P < 0.01, *P < 0.05.

**Figure 10:**
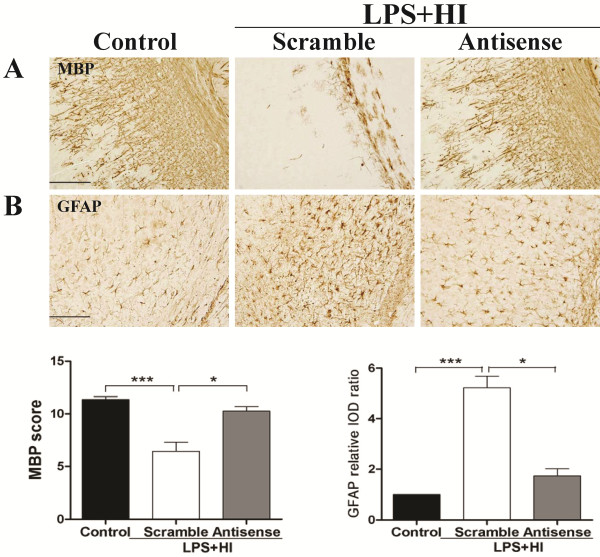
**c-Jun N-terminal kinase (JNK) antisense oligodeoxynucleotide significantly attenuated white matter injury.** Antisense oligodeoxynucleotide (ODN) treatment (n = 8) markedly increased myelin basic protein (MBP) (**A**) and decreased glial fibrillary acidic protein (GFAP) (**B**) expression in the white matter compared with scrambled ODN (n = 8) on P11 after lipopolysaccharide (LPS)-sensitized hypoxic-ischemia (HI) on P2. Scale bar = 200 μm in (A), 100 μm in (B). Values are means ± SEM. ***P < 0.001, *P < 0.05.

We next examined the protective effect of JNK inhibition on white matter injury using JNK antisense ODN. Immunoblotting analyses of the white matter tissue of the LPS + HI group showed that JNK antisense ODN treatment significantly reduced JNK expression at 3, 6 and 12 h post-insult compared to scrambled ODN (Figure 9A). Antisense ODN treatment significantly diminished the numbers of ED1-positive activated microglia, TNF-α immunoreactivities, BBB breakdown and cleaved caspase 3-positive cells in the white matter 24 h post-insult compared to scrambled ODN treatment (Figure 9B). Antisense ODN treatment on P2 in the LPS + HI group also increased MBP expression (Figure 10A) and markedly attenuated astrogliosis (Figure 10B) in the white matter on P11 compared with scrambled ODN (Figure 10A,B).

## Discussion

White matter injury is the major form of brain injury in very preterm infants. The O4-positive oligodendrocyte progenitors, mainly pre-myelinating oligodendrocytes in P2 rat brain, are the major target cells of damage in the white matter of very premature infants [12,13]. In this study, we showed that P2 rat pups had selective white matter injury (hypomyelination and astrogliosis) on P11 after LPS-sensitized HI. White matter injury in the immature brain was associated with early and sustained JNK activation in the microglia, vascular endothelial cells and oligodendrocyte progenitors within 24 h post-insult, and also with upregulation of microglia activation, TNF-α expression, BBB leakage, and endothelial cell and oligodendroglial apoptosis 24 h post-insult. Pharmacological or genetic inhibition of JNK reduced microglia activation, TNF-α expression, BBB damage and oligodendrocyte progenitor apoptosis, and protected against white matter injury after LPS-sensitized HI. These findings suggest that JNK signaling is the shared pathway linking neuroinflammation, vascular endothelial cell damage and BBB breakdown, and apoptosis of oligodendroglial precursor cells in the white matter injury of the immature brain.

Very preterm infants experience various HI and infectious insults during the neonatal period. Infection may predispose to, or act in concert with, HI in premature infants. Previous studies show that increased systemic cytokines in premature infants with chorioamnionitis are associated with hemodynamic disturbance leading to cerebral HI [6,7], whereas co-morbid chorioamnionitis and placental perfusion defect put preterm infants at higher risk of abnormal neurological outcomes than either insult alone [8]. Our previous study using the P2 rat pup model to mimic brain injury in very preterm infants demonstrated that selective white matter injury could be induced by the combination of LPS and HI rather than by LPS exposure or HI alone [14]. We found that low-dose LPS upregulated JNK activation in the white matter without causing tissue damage. In contrast, LPS + HI elicited early and prolonged activation of JNK and resulted in white matter injury. Studies investigating the mechanisms of LPS sensitization show early upregulation of genes associated with stress-induced inflammatory responses in the immature brain several hours after LPS exposure [42,43], and the priming effect may contribute to increased vulnerability of the immature brain to HI following LPS exposure.

The key features of LPS-sensitized HI white matter injury in the immature brain include: (1) neuroinflammation, manifested as activation of microglia and upregulation of TNF-α; (2) vascular endothelial cell damage and BBB breakdown; and (3) apoptosis of O4-positive oligodendrocyte progenitors [1,2,14]. Although previous studies have shown that LPS and/or HI induced any one of the key features of injury in the neonatal rodent brain [11,40,44], very few studies have examined the three pathogenic mechanisms as an oligodendrovascular unit in the white matter, particularly in the immature P2 rat brain. In the white matter, microglia, vascular endothelial cells and oligodendrocyte progenitors are closely knitted together with reciprocal interactions. In physiological conditions, vascular endothelial cells are the kernel of BBB and supply oxygen and nutrients from the blood stream to adjacent brain parenchyma. Both endothelial and various neural cells can secrete angioneurins to mutually facilitate vascular and neural development [45]. The survival, proliferation and differentiation of oligodendrocyte progenitors are regulated by growth factors released from neural cells [1,46]. During detrimental insults, the activated microglia may trigger a cascade of reactions, via proinflammatory cytokines, leading to destroyed BBB damage and cell apoptosis in the white matter. The damaged microvessels may further recruit activated leukocytes through the injured BBB and cause sustained activation of microglia, which in turn causes further damage in the white matter [21]. Therefore, to achieve effective therapies for white matter injury is to protect the entire “oligodendrovascular unit” through blockade of the common signal transduction linking neuroinflammation, BBB damage and cell apoptosis.

Activated microglia play a central role as a converging point for upstream HI/inflammation and downstream cytotoxicity in the pathogenesis of white matter injury in the immature brain [20]. In this study, the findings that LPS-sensitized HI contributes to JNK activation and the nuclear translocation of the downstream molecule c-Jun in the microglia further highlight the neuroinflammatory role of microglia in the white matter injury. The transcription factor c-Jun subsequently leads to pro-inflammatory cytokine production, identified in this study as TNF-α expression in microglia. The increase of TNF-α immunoreactivities in the white matter corresponds to the region-specific activation of microglia in this P2 rat pup model of white matter injury. The microglia-derived TNF-α may not only exert cytotoxic effects on oligodendrocyte progenitors and endothelial cells, but also facilitate prolonged microglial activation via activation of JNK synthesis in an autocrine loop in the oligodendrovascular unit [47,48].

The BBB acts as a pivotal interface for central- and peripheral-driven inflammatory processes in brain injury. In this neonatal rat model, systemic LPS exposure plus cerebral HI insult triggered BBB disruption and selective white matter injury. We used extravasation of IgG as an index of BBB damage. After LPS + HI, the extravascular IgG immunoreactivity in the white matter could be observed at the cellular as well as the parenchymal level. IgG entry into neural cells after brain injury has been described in studies using immunostaining [49-51]. Glial cells can rapidly take up plasma proteins from the extracellular space of the injured brain through endocytosis, and Fc-receptors on reactive microglia can trap IgG in the tissue and thus facilitate its phagocytic activity [50,51]. The vulnerability of BBB in the white matter correlated with the region-specific activation of microglia. JNK-positive activated microglia released TNF-α, which may contribute to BBB breakdown through upregulation of matrix metalloproteinase-9 [52] or via triggering death signaling in vascular endothelial cells [53]. The cytotoxic effects of TNF-α on endothelial cells may be mediated directly through formation of a death-inducing signaling complex or indirectly via JNK activation [48]. We demonstrated that, after insult, vascular endothelial cells had both p-JNK and cleaved caspase 3 expression, and p-JNK-positive cells co-expressed cleaved caspase 3. The findings suggest the role of JNK signaling in vascular endothelial cell apoptosis after LPS-sensitized HI.

A noteworthy finding in this study was that many p-JNK-positive cells surrounded, or were attached to, the microvessels in the white matter after insult. These p-JNK-positive cells may be exogenous leukocytes infiltrating through the disrupted BBB, or endogenous brain cells such as microglia. The activated leukocytes may diminish the effectiveness of the immature BBB and contribute to sustained BBB disruption by enhancing matrix metalloproteinase-9 activity [54,55]. In addition, the leukocytes migrating into the brain may activate microglia, which in turn further damage the BBB and secrete chemokines to attract more activated leukocytes into the white matter [21,56]. The BBB disruption by leukocytes and microglia may also be mediated through JNK/TNF-α signaling [31,52]. Therefore the increases of BBB permeability in the white matter may act in concert with activated microglia to worsen white matter injury through leukocyte recruitment into the brain [21].

Oligodendrocyte precursor cells are the end-target of white matter injury in the oligodendrovascular unit, and exhibit maturation-dependent vulnerability [1,2,20]. Pre-myelinating oligodendrocytes display greater susceptibility to pro-inflammatory cytokines, oxidative damage and glutamate excitotoxicity than do mature oligodendrocytes [1,20]. Our study showed that O4-positive oligodendrocyte progenitors had sustained JNK activation after insult, and were the major cells expressing cleaved caspase 3 apoptotic markers in the white matter. The co-localization of p-JNK and cleaved caspase 3 in the white matter further implicated the key role of JNK signaling in triggering death events in oligodendrocyte precursor cells. In addition to cell death, surviving oligodendrocyte progenitors may be deterred from proliferation and differentiation by microglial activation and reactive astrocytes [57]. Our findings of reactive astrogliosis and hypomyelination on P11 after LPS + HI reflected the effects of neuroinflammation and impairment of oligodendroglial maturation.

The upstream molecule or signaling pathway that leads to JNK activation in the oligodendrovascular unit of the white matter in the very immature brain remains unclear. Common to both ischemia and inflammation is the production of reactive oxygen and nitrogen species (ROS/RNS), in particular nitric oxide. Nitric oxide production in excess can be detrimental, particularly in the presence of ROS, which are known to be associated with oligodendrocyte death and white matter injury in preterm infants [58-61]. Autopsy studies in preterm infants with periventricular white matter injury have demonstrated protein nitration and lipid peroxidation in pre-myelinating oligodendrocytes [60,61]. An animal experiment showed that the free radical scavenging agent N-acetylcysteine effectively protected against LPS-sensitized HI brain injury in neonatal rats [11]. These findings suggest a role for ROS/RNS in the pathogenesis of white matter injury. Studies have also demonstrated that the synergistic effect of LPS and HI activated microglia to produce ROS/RNS [11,20], leading to prolonged JNK activation which in turn facilitated TNF-α synthesis and more ROS/RNS accumulation in a positive feedback loop [62,63]. These studies showed that JNK signaling is a key modulator in cell death mediated by ROS/RNS [63]. Activated microglia may contribute to BBB breakdown and exert cytotoxicity to endothelial cells and oligodendrocyte progenitors through both JNK-TNF-α and ROS/RNS pathways [20,52,59]. The pre-myelinating oligodendrocytes are particularly more vulnerable to oxidative and nitrosative injury than mature oligodendrocytes due to impaired antioxidant defenses and susceptibility to glutamate excitotoxicity [1,20]. Exuberant expression of calcium-permeable glutamate receptors and overexpression of glutamate transporters in the immature brain give rise to the maturation-dependent vulnerability of pre-myelinating oligodendrocytes to glutamate excitotoxicity [1,20]. During detrimental insults, elevated extracellular glutamate facilitates Ca^2+^ influx through glutamate receptors in oligodendrocyte progenitors, and thus induces ROS/RNS production which further augments JNK activation-mediated apoptosis [20,64]. Therefore, LPS-sensitized HI may damage the oligodendrovascular unit in the immature brain via a self-potentiating loop of ROS/RNS-JNK-TNF-α signaling, which leads to sustained microglial activation, BBB disruption and oligodendroglial apoptosis in a vicious cycle. Further study is needed to address the role of ROS/RNS as the upstream mechanism of JNK activation in the oligodendrovascular unit of the white matter injury of the immature brain after LPS and HI injury.

Previous studies have shown that JNK inhibitors exerted neuroprotective effects against focal or global ischemic injury in adult rodent models of stroke [25,27,28,65], and JNK3 knock-out mice were protected from HI brain injury [26,66]. Using both pharmacological and genetic approaches, this study demonstrated that inhibition of JNK activation significantly reduced neuroinflammation and preserved the oligodendrovascular unit integrity, and thus protected against white matter injury after LPS-sensitized HI in the immature brain.

## Conclusions

In this P2 rat pup model of selective white matter injury, JNK signaling was upregulated in the white matter after LPS-sensitized HI, and acted as the shared pathway integrating neuroinflammation, BBB breakdown and cell apoptosis in the oligodendrovascular unit. A proposed diagram ( Additional file 2; Figure 2) is provided to show that in the three major cells within the oligodendrovascular unit - microglia, endothelial cells and oligodendrocyte progenitors - JNK and TNF-α may potentiate with each other in an autocrine or paracrine pattern to aggravate white matter injury. Suppression of JNK activation, either with the pharmacological inhibitor or by genetic knockdown of the JNK gene, effectively protected against LPS-sensitized HI white matter injury in the immature brain. JNK signaling may emerge as a potential therapeutic target for white matter injury in very preterm infants.

## Abbreviations

BBB, Blood–brain barrier; ED1, Microglia marker; GFAP, Glial fibrillary acidic protein; HI, Hypoxic-ischemia; Ig, Immunoglobulin; IOD, Integrated optical density; JNK, c-Jun N-terminal kinases; LPS, Lipopolysaccharide; MBP, Myelin basic protein; NS, Normal saline; ODN, Oligodeoxynucleotides; P, Postpartum; PBS, Phosphate-buffered saline; p-JNK, Phospho-c-Jun N-terminal kinases; RNS, Reactive nitrogen species; ROS, Reactive oxygen species; TNF, Tumor necrosis factor.

## Competing interests

The authors declare that they have no competing interests.

## Authors’ contributions

LWW and CCH participated in the design and execution of the study and performed the statistical analysis. YFT provided continuous intellectual input, and evaluated and interpreted the data. LWW, YFT and CJH provided technique support for animal preparation and carried out the immunoassays. CCH conceived, designed and coordinated the project, and drafted the manuscript. All authors have read and approved the final manuscript.

## Supplementary Material

Additional file 1 **Figure 1.** Neuropathological examinations in the lipopolysaccharide (LPS)-treated group on P11 demonstrated no evident (A) cortical neuronal injury by Nissl staining or (B) white matter injury by myelin basic protein (MBP) staining. (C) Immunohistochemistry at 24 h post-insult also did not show significant increases of ED1-positive microglia and IgG extravasation in the white matter of the LPS-treated group. (D) Immunoblotting of the white matter showed increased phosphor-c-Jun N-terminal kinase **(**p-JNK) expression at 24 h post-LPS. Scale bar = 200 μm for MBP, and 100 μm for the others. Click here for file

Additional file 2 **Figure 2.** A proposed diagram showing the central role of c-Jun N-terminal kinase (JNK) signaling in the pathogenesis of lipopolysaccharide (LPS)-sensitized hypoxic-ischemic (HI) white matter injury in the immature brain. JNK hyperactivation in the oligodendrovascular unit (microglia, microvascular endothelial cells and oligodendrocyte progenitors) post-insult may lead to white matter injury through upregulation of neuroinflammation, blood–brain barrier disruption and oligodendrocyte progenitor apoptosis. Click here for file

## References

[B1] Volpe JJ Brain injury in premature infants: a complex amalgam of destructive and developmental disturbances Lancet Neurol 2009 8 110 124 10.1016/S1474-4422(08)70294-1 19081519 PMC2707149

[B2] Volpe JJ Neurology of the Newborn 2008 5 W.B. Saunders Co, Philadelphia

[B3] Vincer MJ Allen AC Joseph KS Stinson DA Scot H Wood E Increasing prevalence of cerebral palsy among very preterm infants: a population-based study Pediatrics 2006 118 e1621 e1626 10.1542/peds.2006-1522 17074842

[B4] McElrath TF Allred EN Boggess KA Kuban K O’Shea TM Paneth N Leviton A Maternal antenatal complications and the risk of neonatal cerebral white matter damage and later cerebral palsy in children born at an extremely low gestational age Am J Epidemiol 2009 170 819 828 10.1093/aje/kwp206 19713285 PMC2765357

[B5] Stoll BJ Hansen NI Adams-Chapman I Fanaroff AA Hintz SR Vohr B Higgins RD Neurodevelopmental and growth impairment among extremely low-birth-weight infants with neonatal infection JAMA 2004 292 2357 2365 10.1001/jama.292.19.2357 15547163

[B6] Yanowitz TD Jordan JA Gilmour CH Towbin R Bowen A Roberts JM Brozanski BS Hemodynamic disturbances in premature infants born after chorioamnionitis: association with cord blood cytokine concentrations Pediatr Res 2002 51 310 316 10.1203/00006450-200203000-00008 11861935

[B7] Tsuji M Saul JP Plessis A Eichenwald E Sobh J Crocker R Volpe JJ Cerebral intravascular oxygenation correlates with mean arterial pressure in critically ill premature infants Pediatrics 2000 106 625 632 10.1542/peds.106.4.625 11015501

[B8] Kaukola T Herva R Perhomma M Paakko E Kingsmore S Vainionpaa L Hallman M Population cohort associating chorioamnionitis, cord inflammatory cytokines and neurological outcome in very preterm, extremely low birth weight infants Pediatr Res 2006 59 478 483 10.1203/01.pdr.0000182596.66175.ee 16492993

[B9] Lehnardt S Massillon L Follett P Jensen FE Ratan R Rosenberg PA Volpe JJ Vartanian T Activation of innate immunity in the CNS triggers neurodegeneration through a Toll-like receptor 4-dependent pathway PNAS 2003 100 8514 8519 10.1073/pnas.1432609100 12824464 PMC166260

[B10] Eklind S Mallard C Leverin AL Gilland E Blomgren K Mattsby-Baltzer I Hagberg H Bacterial endotoxin sensitizes the immature brain to hypoxic-ischemic injury Eur J Neurosci 2001 13 1101 1106 10.1046/j.0953-816x.2001.01474.x 11285007

[B11] Wang X Svedin P Nie C Lapatto R Zhu C Gustavsson M Sandberg M Karlsson JO Romero R Hagberg H Mallard C N-acetylcysteine reduces lipopolysaccharide-sensitized hypoxic-ischemic brain injury Ann Neurol 2007 61 263 271 10.1002/ana.21066 17253623

[B12] Back SA Luo NL Borenstein NS Levin JM Volpe JJ Kinney HC Late oligodendrocyte progenitors coincide with the developmental window of vulnerability for human perinatal white matter injury J Neurosci 2001 21 1302 1312 11160401 10.1523/JNEUROSCI.21-04-01302.2001 PMC6762224

[B13] Craig A Luo NL Beardsley DJ Wingate-Pearse N Walker DW Hohimer AR Back SA Quantitative analysis of perinatal rodent oligodendrocyte lineage progression and its correlation with human Exp Neurol 2003 181 231 240 10.1016/S0014-4886(03)00032-3 12781996

[B14] Wang LW Chang YC Lin CY Hong JS Huang CC Low-dose lipopolysaccharide selectively sensitizes hypoxia-ischemia-induced white matter injury in the immature brain Pediatr Res 2010 68 41 47 10.1203/PDR.0b013e3181df5f6b 20351655 PMC3608684

[B15] del Zoppo GJ Stroke and neurovascular protection N Engl J Med 2006 354 553 555 10.1056/NEJMp058312 16467542

[B16] Chew LJ Takanohashi A Bell M Microglia and inflammation: impact on developmental brain injuries Ment Retard Dev Disabil Res Rev 2006 12 105 112 10.1002/mrdd.20102 16807890

[B17] Muramatsu K Fukuda A Togari H Wada Y Nishino H Vulnerability to cerebral hypoxic-ischemic insult in neonatal but not in adult rats is in parallel with disruption of the blood–brain barrier Stroke 1997 28 2281 2288 10.1161/01.STR.28.11.2281 9368577

[B18] Tu YF Tsai YS Wang LW Wu HC Huang CC Ho CJ Overweight worsens apoptosis, neuroinflammation and blood–brain barrier damage after hypoxic ischemia in neonatal brain through JNK hyperactivation J Neuroinflammation 2011 8 40 54 10.1186/1742-2094-8-40 21518436 PMC3090337

[B19] Tu YF Lu PJ Huang CC Moderate dietary restriction reduces p53-mediated neurovascular damage and microglia activation after hypoxic ischemia in neonatal brain Stroke 2012 43 491 498 10.1161/STROKEAHA.111.629931 22076005

[B20] Khwaja O Volpe JJ Pathogenesis of cerebral white matter injury of prematurity Arch Dis Child Fetal Neonatal Ed 2008 93 F153 F161 18296574 10.1136/adc.2006.108837 PMC2569152

[B21] Dammann O Durums S Leviton A Do white cells matter in white matter damage? Trends Neurosci 2001 24 320 324 10.1016/S0166-2236(00)01811-7 11356502

[B22] Manning AM Davis RJ Target JNK for therapeutic benefit: from Junk to gold? Nat Rev Drug Discov 2003 2 554 565 10.1038/nrd1132 12815381

[B23] Cao J Semenova MM Solovyan VT Han J Coffey ET Courtney MJ Distinct requirements for p38alpha and c-Jun N-terminal kinase stress-activated protein kinases in different forms of apoptotic neuronal death J Biol Chem 2004 279 35903 35913 10.1074/jbc.M402353200 15192112

[B24] Varfolomeev EE Ashkenazi A Tumor necrosis factor: an apoptosis JuNKie? Cell 2004 116 491 497 10.1016/S0092-8674(04)00166-7 14980217

[B25] Gao Y Signore AP Yin W Cao G Yin XM Sun F Luo Y Graham SH Chen J Neuroprotection against focal ischemic brain injury by inhibition of c-Jun N-terminal kinase and attenuation of the mitochondrial apoptosis-signaling pathway J Cereb Blood Flow Metab 2005 25 694 712 10.1038/sj.jcbfm.9600062 15716857

[B26] Kuan CY Whitmarsh AJ Yang DD Liao G Schloemer AJ Dong C Bao J Banasiak KJ Haddad GG Flavell RA Davis RJ Rakic P A critical role of neural-specific JNK3 for ischemic apoptosis PNAS 2003 100 15184 15189 10.1073/pnas.2336254100 14657393 PMC299947

[B27] Guan QH Pei DS Zong YY Xu TL Zhang GY Neuroprotection against ischemic brain injury by a small peptide inhibitor of c-Jun N-terminal kinase via nuclear and non-nuclear pathways Neurosci 2006 139 609 627 10.1016/j.neuroscience.2005.11.067 16504411

[B28] Guan QH Pei DS Liu XM Wang XT Xu TL Zhang GY Neuroprotection against ischemic brain injury by SP600125 via suppressing the extrinsic and intrinsic pathways of apoptosis Brain Res 2006 1092 36 46 10.1016/j.brainres.2006.03.086 16674927

[B29] Uesugi M Nakajima K Tohyama Y Kohsaka S Kurihara T Nonparticipation of nuclear factor kappa B in the signaling cascade of c-Jun N-terminal kinases and p38 mitogen activated protein kinase-dependent tumor necrosis factor-alpha induction in lipopolysaccharide-stimulated microglia Brain Res 2006 1073 48 59 16457791 10.1016/j.brainres.2005.12.043

[B30] Deng YY Lu J Sivakumar V Ling EA Kaur C Amoeboid microglia in the periventricular white matter induce oligodendrocyte damage through expression of proinflammatory cytokines via MAP kinase signaling pathway in hypoxic neonatal rats Brain Pathol 2008 18 387 400 10.1111/j.1750-3639.2008.00138.x 18371179 PMC8095524

[B31] Yatsusshige H Ostrowski RP Tsubokawa T Colohan A Zhang JH Role of c-Jun N-terminal Kinase in early brain injury after subarachnoid hemorrhage J Neurosci Res 2007 85 1436 1448 10.1002/jnr.21281 17410600

[B32] Karahashi H Michelsen KS Arditi M Lipopolysaccharide-induced apoptosis in transformed bovine brain endothelial cells and human dermal microvessel endothelial cells: the role of JNK J Immunol 2009 182 7280 7286 10.4049/jimmunol.0801376 19454725 PMC3057198

[B33] Pirianov G Jesurasa A Mehmet H Developmentally regulated changes in c-Jun N-terminal kinase signaling determine the apoptotic response of oligodendrocyte lineage cells Cell Death Differ 2006 13 531 533 10.1038/sj.cdd.4401805 16322755

[B34] Repici M Centeno C Tomasi S Forloni G Bonny C Vercelli A Borsello T Time-course of c-Jun N-terminal kinase activation after cerebral ischemia and effect of D-JNKI1 on c-Jun and caspase-3 activation Neuroscience 2007 150 40 49 10.1016/j.neuroscience.2007.08.021 17900813

[B35] Herdegen T Claret FX Kallunki T Martin-Villalba A Winter C Hunter T Karin M Lasting N-terminal phosphorylation of c-Jun and activation of c-Jun N-terminal kinases after neuronal injury J Neurosci 1998 18 5124 5135 9651196 10.1523/JNEUROSCI.18-14-05124.1998 PMC6793486

[B36] Chang YC Huang CC Hung PL Huang HM Rolipram, a phosphodiesterase type IV inhibitor, exacerbates periventricular white matter lesions in rat pups Pediatr Res 2008 64 234 239 10.1203/PDR.0b013e31817cfc87 18437099

[B37] Carboni S Hiver A Szyndralewiez C Gaillard P Gotteland JP Vitte PA AS601245 (1,3-Benzothiazol-2-yl (2-{[2-(3-pyridinyl) ethyl] amino}-4 pyrimidinyl) Acetonitrile): a c-Jun NH2-terminal protein kinase inhibitor with neuroprotective properties J Pharmacol Exp Ther 2004 310 25 32 10.1124/jpet.103.064246 14988419

[B38] Lin HY Wu CL Huang CC The Akt-endothelial nitric oxide synthase pathway in lipopolysaccharide preconditioning-induced hypoxic-ischemic tolerance in the neonatal rat brain Stroke 2010 41 1543 1551 10.1161/STROKEAHA.109.574004 20508195

[B39] Paxinos G Watson C The rat brain in stereotaxic coordinates 1986 Academic, New York 10.1016/0165-0270(80)90021-7 6110810

[B40] Svedin P Hagberg H Savman K Zhu C Mallard C Matrix metalloproteinase-9 gene knock-out protects the immature brain after cerebral hypoxia-ischemia J Neurosci 2007 27 1511 1518 10.1523/JNEUROSCI.4391-06.2007 17301159 PMC6673738

[B41] Lin HY Huang CC Chang KF Lipopolysaccharide preconditioning reduces neuroinflammation against hypoxic ischemia and provides long-term outcome of neuroprotection in neonatal rat Pediatr Res 2009 66 254 259 10.1203/PDR.0b013e3181b0d336 19531979

[B42] Eklind S Hagberg H Wang X Savman K Leverin AL Hedtjarn M Mallard C Effect of lipopolysaccharide on global gene expression in the immature rat brain Pediatr Res 2006 60 161 168 10.1203/01.pdr.0000228323.32445.7d 16864697

[B43] Wang X Stridh L Li W Dean J Elmgren A Gan L Eriksson K Hagberg H Mallard C Lipopolysaccharide sensitizes neonatal hypoxic-ischemic brain injury in a MyD88-dependent mammer J Immunol 2009 183 7471 7477 10.4049/jimmunol.0900762 19917690

[B44] Fan LW Mitchell HJ Tien LT Zheng B Pang Y Rhodes PG Cai Z α-phenyl-n-tert-butyl-nitrone reduces lipopolysaccharide-induced white matter injury in the neonatal rat brain Dev Neurobiol 2008 68 365 378 10.1002/dneu.20591 18161853

[B45] Zacchigna S Lambrechts D Carmeliet P Neurovascular signaling defects in neurodegeneration Nat Rev Neurosci 2008 9 169 181 10.1038/nrn2336 18253131

[B46] Back SA Volpe JJ Cellular and molecular pathogenesis of periventricular white matter injury Ment Retard Dev Disabil Res Rev 1997 3 96 107 10.1002/(SICI)1098-2779(1997)3:1<96::AID-MRDD12>3.0.CO;2-M

[B47] Kuno R Wang J Kawanokuchi J Takeuchi H Mizuno T Suzumura A Autocrine activation of microglia by tumor necrosis factor-α J Neuroimmunol 2005 162 89 96 10.1016/j.jneuroim.2005.01.015 15833363

[B48] Baud V Karin M Signal transduction by tumor necrosis factor and its relatives Trends Cell Biol 2001 9 372 377 11514191 10.1016/s0962-8924(01)02064-5

[B49] Remmers M Schmidt-Kastner R Belayev L Lin B Busto R Ginsberg MD Protein extravasation and cellular uptake after high-dose human-albumin treatment of transient focal cerebral ischemia in rats Brain Res 1999 827 237 242 10.1016/S0006-8993(99)01304-9 10320717

[B50] Del Bigio MR Deck JHN Davidson GS Glial swelling with eosinophilia in human post-mortem brains: a change indicative of plasma extravasation Acta Neuropathol 2000 100 688 694 10.1007/s004010000236 11078221

[B51] Jensen MB Finsen B Zimmer J Morphological and immunophenotypic microglial changes in the denervated fascia dentata of adult rats: Correlation with blood–brain barrier damage and astroglial reactions Exp Neurol 1997 143 103 116 10.1006/exnr.1996.6337 9000449

[B52] Rosenberg GA Matrix metalloproteinases in neuroinflammation Glia 2002 39 279 291 10.1002/glia.10108 12203394

[B53] Lucas R Garcia I Donati YRA Hribar M Mandriota SJ Giroud C Buurman WA Fransen L Suter PM Nunez G Pepper MS Grau GE Both TNF receptors are required for direct TNF-mediated cytotoxicity in microvascular endothelial cells Eur J Immunol 1998 28 3577 3586 10.1002/(SICI)1521-4141(199811)28:11<3577::AID-IMMU3577>3.0.CO;2-# 9842900

[B54] De Boer AG Breimer DD Cytokines and blood–brain barrier permeability Prog Brain Res 1998 115 425 451 9632945 10.1016/s0079-6123(08)62045-2

[B55] McColl BW Rothwell NJ Allan SM Systemic inflammation alters the kinetics of cerebrovascular tight junction disruption after experimental stroke in mice J Neurosci 2008 28 9451 9462 10.1523/JNEUROSCI.2674-08.2008 18799677 PMC6671112

[B56] D’Mello C Le T Swain MG Cerebral microglia recruit monocytes into the brain in response to tumor necrosis factor-α signaling during peripheral organ inflammation J Neurosci 2009 29 2089 2102 10.1523/JNEUROSCI.3567-08.2009 19228962 PMC6666330

[B57] Volpe JJ Systemic inflammation, oligodendroglial maturation and encephalopathy of prematurity Ann Neurol 2011 70 525 529 10.1002/ana.22533 22028217

[B58] Baud O Li J Zhang Y Neve RL Volpe JJ Rosenberg PA Nitric oxide-induced cell death in developing oligodendrocytes is associated with mitochondrial dysfunction and apoptosis-inducing factor translocation Eur J Neurosci 2004 20 1713 1726 10.1111/j.1460-9568.2004.03616.x 15379992

[B59] Li J Baud O Vartanian T Volpe JJ Rosenberg PA Peroxynitrite generated by inducible nitric oxide synthase and NADPH oxidase mediates microglial toxicity to oligodendrocytes Proc Natl Acad Sci USA 2005 102 9936 9941 10.1073/pnas.0502552102 15998743 PMC1174990

[B60] Haynes RL Folkerth RD Keefe RJ Sung I Swzeda LI Rosenberg PA Volpe JJ Kinney HC Nitrosative and oxidative injury to premyelinating oligodendrocytes in periventricular leukomalacia J Neuropathol Exp Neurol 2003 62 441 450 12769184 10.1093/jnen/62.5.441

[B61] Back SA Luo NL Mallinson RA O’Malley JP Wallen LD Frei B Morrow JD Petito CK Roberts CT Murdoch GH Montine TJ Selective vulnerability of preterm white matter to oxidative damage defined by F2-isoprostanes Ann Neurol 2005 58 108 120 10.1002/ana.20530 15984031

[B62] Kamata H Honda S Maeda S Chang L Hirata H Karin M Reactive oxygen species promote TNF-α-induced death and sustained JNK activation by inhibiting MAP kinase phosphatases Cell 2005 120 649 661 10.1016/j.cell.2004.12.041 15766528

[B63] Shen HM Liu ZG JNK signaling is a key modulator in cell death mediated by reactive oxygen and nitrogen species Free Radical Biol Med 2006 40 928 939 10.1016/j.freeradbiomed.2005.10.056 16540388

[B64] Liu HN Giasson BI Mushynski WE Almazan G AMPA receptor-mediated toxicity in oligodendrocyte progenitors involves free radical generation and activation of JNK, calpain and caspase 3 J Neurochem 2002 82 398 409 10.1046/j.1471-4159.2002.00981.x 12124441

[B65] Borsello T Clarke PGH Hirt L Vercelli A Repici M Schorderet DF Bogousslavsky J Bonny C A peptide inhibitor of c-Jun N-terminal kinase protects against excitotoxicity and cerebral ischemia Nat Med 2003 9 1180 1186 10.1038/nm911 12937412

[B66] Pirianov G Brywe K Mallard C Edwards AD Flavell RA Hagberg H Mehmet H Deletion of the c-Jun N-terminal kinase 3 gene protects neonatal mice against cerebral hypoxic-ischemic injury J Cereb Blood Flow Metab 2007 27 1022 1032 17063149 10.1038/sj.jcbfm.9600413

