# Supplementary figures and images for: JNK signaling is the shared pathway linking neuroinflammation, blood–brain barrier disruption, and oligodendroglial apoptosis in the white matter injury of the immature brain
Source: J Neuroinflammation. 2012 Jul 17;9:175. doi: 10.1186/1742-2094-9-175 (PMC3414763; doi:10.1186/1742-2094-9-175)

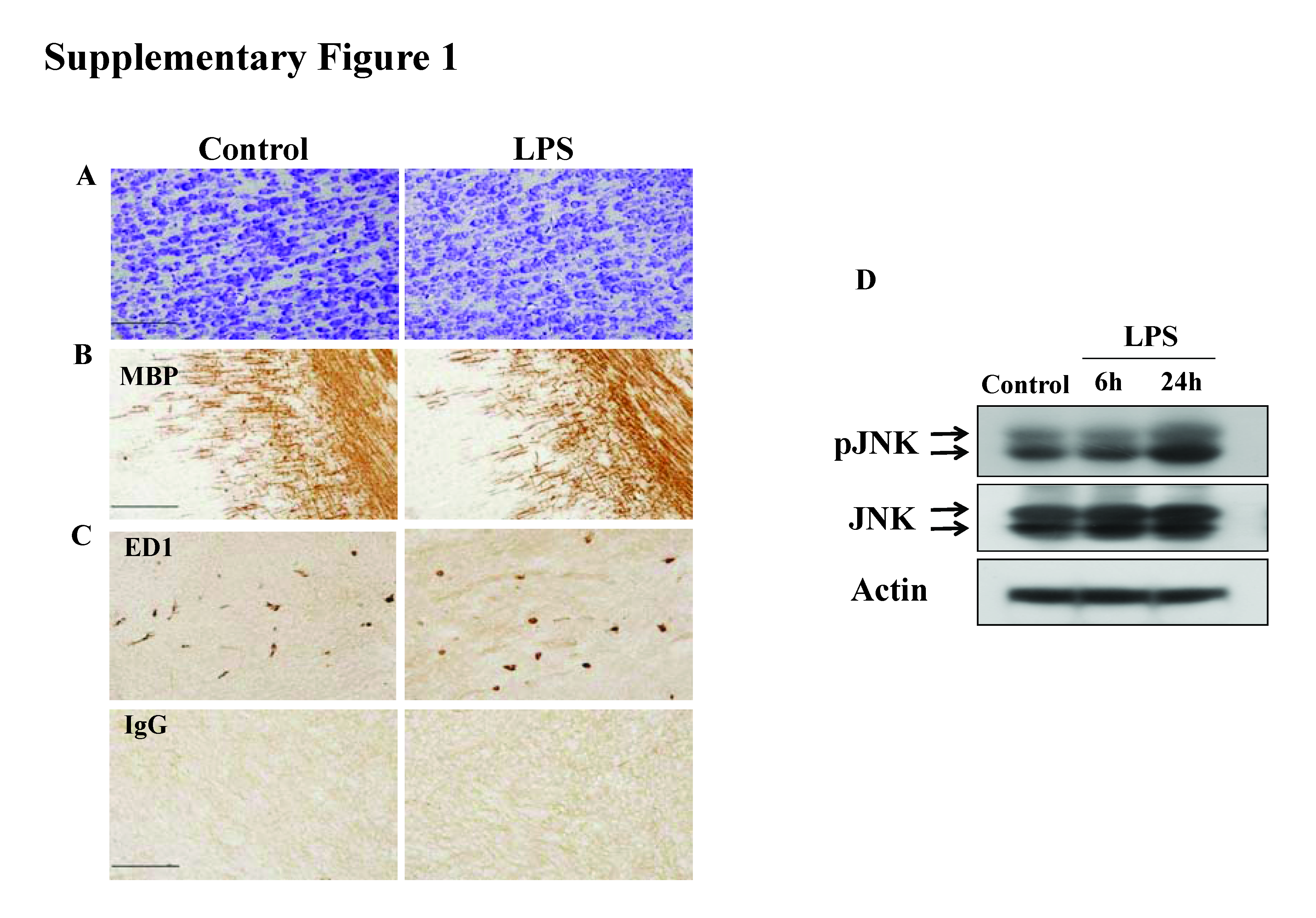

Supplement: Additional file 1 — Figure 1. Neuropathological examinations in the lipopolysaccharide (LPS)-treated group on P11 demonstrated no evident (A) cortical neuronal injury by Nissl staining or (B) white matter injury by myelin basic protein (MBP) staining. (C) Immunohistochemistry at 24 h post-insult also did not show significant increases of ED1-positive microglia and IgG extravasation in the white matter of the LPS-treated group. (D) Immunoblotting of the white matter showed increased phosphor-c-Jun N-terminal kinase (p-JNK) expression at 24 h post-LPS. Scale bar = 200 μm for MBP, and 100 μm for the others. [file 1742-2094-9-175-S1.tiff]
